# Preparation of Few-Micron-Thick Free-Standing Au-Nanorod/UDMA-TEGDMA Nanocomposite Films by Using PVA Sacrificial Layers

**DOI:** 10.3390/polym17101391

**Published:** 2025-05-19

**Authors:** Nóra Tarpataki, Andrea Keczánné-Üveges, Melinda Szalóki, Attila Bonyár

**Affiliations:** 1 Department of Electronics Technology, Faculty of Electrical Engineering and Informatics, Budapest University of Technology and Economics, H1111 Budapest, Hungary; tarpatakin@edu.bme.hu; 2 Department of Biomaterials and Prosthetic Dentistry, Faculty of Dentistry, University of Debrecen, H4032 Debrecen, Hungary; auveges@dental.unideb.hu (A.K.-Ü.); szaloki.melinda@dental.unideb.hu (M.S.); 3 HUN-REN Wigner Research Centre for Physics, H1525 Budapest, Hungary

**Keywords:** nanocomposites, PVA, UDMA-TEGDMA, spin coating

## Abstract

A method to prepare free-standing, few-micron-thick films from a dental photopolymer resin, namely UDMA-TEGDMA in a 3:1 weight ratio, doped with gold nanorods, is presented. The method is based on a sandwich structure consisting of a 4 μm thick PVA sacrificial layer, the Au-nanorod/UDMA-TEGDMA nanocomposite layer, and glycerol, all spin-coated sequentially onto a glass slide. Glycerol serves as a cover layer to shut out oxygen during photopolymerization, while the water-soluble PVA enables the subsequent detachment of the nanocomposite film by simple immersion into a liquid bath. Layer thicknesses were controlled by profilometry, while the presence of homogeneously dispersed gold nanorods was confirmed by optical spectroscopy and dark-field optical microscopy. A total of five similar spin-coating scenarios were tested, out of which two approaches produced positive results, with final nanocomposite layer thicknesses in the 2.5–4 μm range, which is smaller than the usual thickness of the oxygen inhibition layer (OIL) commonly present in these types of resins. Optimization of these technological processes and parameters to control film thickness and consistency is discussed in detail.

## 1. Introduction

One of the most common methods for depositing thin polymer layers on substrates is spin coating, which allows for the production of uniform films. This method typically uses polymer solutions to form thin films under the influence of centripetal force, followed by the removal of the solvent [1]. However, rapid evaporation of the solvent can result in unreliable and fragile membranes [2], so solvent-free film formation, i.e., direct application of monomers and in situ polymerization, can be advantageous. The properties of the resulting polymer film can be designed by the monomer and the polymerization reaction conditions. Copolymer films can provide additional favorable physical and chemical properties by combining the advantageous properties of the monomers. The basic conditions for selecting monomers are their compatibility and excellent miscibility so that phase separation does not occur during spin coating.

One optional process for preparing a thin polymer layer is the free radical polymerization of a resin. This bulk polymerization combined with a photoinitiator system results in a predictable and quasi-controllable solidification process. Some of the most suitable and usable molecules for this purpose are methacrylates. The urethane dimethacrylate (UDMA) and triethylene glycol dimethacrylate (TEGDMA) monomers are well known and frequently used in dentistry [3,4]. The UDMA and TEGDMA dimethacrylates form a three-dimensional polymer network upon polymerization that resists the solvents and contributes to thin film strength. TEGDMA has high reactivity that minimizes the residual monomers in the formed polymer [5]. Due to the urethane linkage of UDMA, it provides a certain toughness [6]. By the combination of TEGDMA and UDMA monomers in a free radical polymerization, a water-resistant photopolymerized thin layer can be prepared in one step [7].

The drawback of this free radical polyreaction is oxygen, which inhibits the polymerization and results in the formation of an oxygen inhibition layer (OIL) on the uppermost surface of the cured thin polymer layer [8,9]. Based on previous studies, the thickness of the oxygen inhibition layer ranges from 4 to 40 µm [10,11,12]. This thickness depends on many factors, like monomer chemistry, free radical concentration, and oxygen consumption rate. The thickness of the OIL can be reduced by photoinitiator concentration and increased light curing (intensity or curing duration) [13,14]. Increased photoinitiator concentration previously resulted in a less durable and strong crosslinked polymer due to the shorter polymer chain formation [8]. Therefore, preparing a dimethacrylate-based layer in a few microns (which overlaps with the usual thickness of the OIL) remains a challenge.

The fabrication of large-area (cm^2^ range) free-standing polymer films can prove challenging, depending on the materials used. Such films are usually prepared by applying sacrificial layers to facilitate the removal of the film from the substrate [15]. However, the surface chemistry of the substrate, the sacrificial layer, and the film should be carefully matched to provide (1) good adhesion during the spin-coating process; (2) good wettability to guarantee uniform spreading for both the adhesion layer and the film material; and (3) selective solubility for subsequent film removal without mechanical damage or chemical contamination. These conditions are not easy to fulfill together.

In this paper, we aim to develop a technology to prepare free-standing few-micron-thick layers of a dimethacrylate-based resin, where the desired thickness of the layer is smaller than the usual dimension of the OIL. In addition to controlling the layer thickness below the OIL range, we demonstrate that layers with a large surface area (several cm^2^) can be removed from the substrate without harm by using a PVA (polyvinyl alcohol) sacrificial layer. For this, a UDMA-TEGDMA mixture is used, where the ester bonds in the TEGDMA provide good chemical compatibility with the OH groups of PVA to guarantee uniform spreading.

The intended application of the thin layers doped with gold nanorods is as target materials in the NanoPlasmonic Laser-Induced Fusion Experiment (NAPLIFE) project [16]. Here, high-energy, femtosecond laser pulses will be used to irradiate the nanocomposite sample [17]. A small target thickness is essential for the high-energy ions to be able to escape from the material and be detected with a Thompson parabola [18].

## 2. Materials and Methods

### 2.1. Preparation of Glass Slides and PVA Solutions

The glass slides were rinsed with ethanol (Merck, Darmstadt, Germany, purity ≥ 99%) and then submitted to plasma treatment (Diener Atto plasma chamber, oxygen plasma, 0.35 mbar, 50% power, 2 min) in order to clean and hydrophilize their surface.

The polymer used for the sacrificial layer was selected based on three criteria: (1) water solubility; (2) ability to properly wet an oxygen plasma-treated glass surface to form a smooth and continuous layer; and (3) amorphous property [19] in order to minimize the surface roughness of the spin-coated film. Accordingly, a 10% *w*/*w* aqueous solution of polyvinyl alcohol (PVA) (Merck, Darmstadt, Germany) (65 kDa, polydispersity: 3.168; quality level: MQ-500) was used, which was prepared by completely dissolving solid PVA in water (24 h, 80 °C, stirring).

### 2.2. Nanocomposite

Dodecanethiol-capped gold nanorods (Au-DDT) with 25 nm short axis and 35 and 85 nm long axis lengths were purchased from Nanopartz Inc., Loveland, CO, USA. For the sake of simplicity, the nanorods with different aspect ratios will be referred to as types 25 × 35 and 25 × 85 in the future.

The photopolymerizable dimethacrylate resin mixture consists of urethane dimethacrylate (UDMA) (Sigma-Aldrich Co., St. Louis, MO, USA; purity ≥ 97%) and triethylene glycol dimethacrylate (TEGDMA) (Sigma-Aldrich Co., St. Louis, MO, USA; purity 95%). The weight ratio of UDMA: TEGDMA was 3:1. Camphorquinone (CQ, 0.2 mm% for weight of monomers; purity 97%) (Sigma-Aldrich Co., St. Louis, MO, USA) was used as the photoinitiator, along with its co-initiator, ethyl-4-dimethylaminobenzoate (EDAB, 0.4 m/m% for weight of monomers; purity ≥ 99%) (Sigma-Aldrich Co., St. Louis, MO, USA). The CQ/EDAB ratio and concentration were maintained constant for all samples. The photoinitiator system was dissolved in the resin matrix by stirring overnight at room temperature. Before the use of nanoparticles, the dispersion was sonicated for 5 min followed by a 2 min vortex. The toluene-dispersed 25 × 35 nm and 25 × 85 nm gold nanorods were added to the UDMA-TEGDMA mixture in 0.1236 m/m%, which resulted in 1.9 × 10^12^ mL^−1^ nominal concentration.

### 2.3. Spin Coating

Although different methods were tested to create thin nanocomposite layers, the first steps were identical in all cases: the 10 *w*/*w*% PVA solution was deposited on the oxygen plasma-treated glass slide and spin-coated (~700 RPM, 60 s). After the layer dried, the mixture containing methacrylate monomers and gold nanoparticles was added to the top.

At this point, different methods were investigated, all incorporating two main parts: spin coating of the mixture and photopolymerization using a standard blue light dental curing lamp (Demetron, VCL401, Middleton, WI, USA) as well as glycerol to cover the sample. This is necessary as oxygen inhibits polymerization and can be excluded in this way. The emission spectrum of this quartz–tungsten–halogen (QTH) lamp can be considered optimal for our photoinitiator. (For respective emission and absorbance spectra, please see [20]). The distance between the lamp and the sample was ~1.5 cm, resulting in a few cm^2^ light spot illuminating the whole area of the mixture. In our previous study, we investigated the in situ polymerization of this mixture and found that with these conditions the layer polymerization saturates in a matter of minutes (see [21]). This was also confirmed with Raman spectroscopy investigations and the determination of the degree of conversion (DC) [17,22]. In these past studies, the layer thickness was 150 ± 5 µm, so we can assume that in this case, with much thinner layers, the polymerization can also be considered complete.

Slight variations in the sequence of steps were introduced to optimize the procedure, as shown in Table 1.

**Figure 1:**
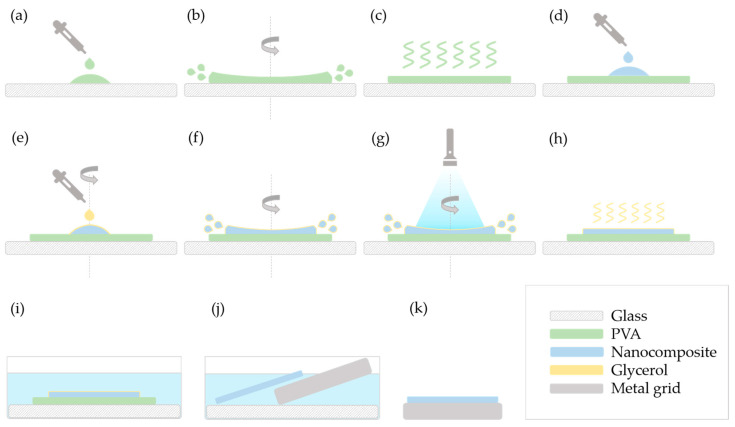
Schematic representation of the spin-coating process, method “A”. Steps: (**a**) deposition of PVA on the glass slide; (**b**) spin coating of PVA; (**c**) drying of the PVA film; (**d**) deposition of the nanocomposite; (**e**) deposition of glycerol during spin-up; (**f**) increasing speed of rotation; (**g**) polymerization of the resin under blue light during spin-off; (**h**) drying of glycerol; (**i**) immersion into water; (**j**) removing the nanocomposite layer with a metal grid; (**k**) the nanocomposite layer on a metal grid.

**Figure 2:**
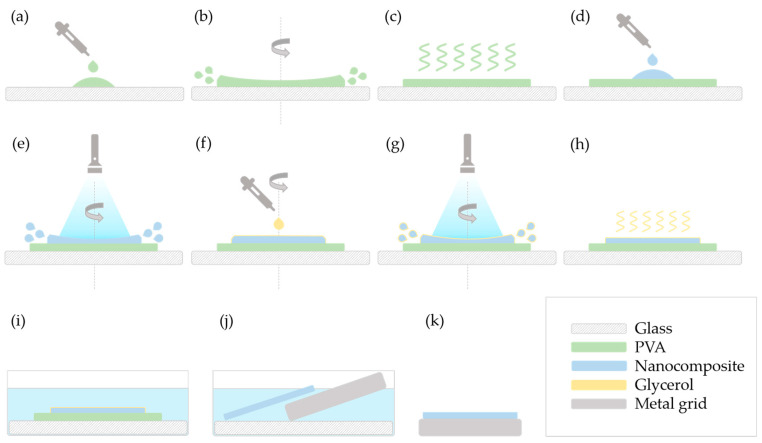
Schematic representation of the spin-coating process, method “E”. Steps: (**a**) deposition of PVA on the glass slide; (**b**) spin coating of PVA; (**c**) drying of the PVA film; (**d**) deposition of the nanocomposite; (**e**) polymerization of the nanocomposite under blue light during spin-off; (**f**) deposition of glycerol; (**g**) polymerization of the nanocomposite under blue light during spin-up of glycerol; (**h**) drying of glycerol; (**i**) immersion into water; (**j**) removing the nanocomposite layer with a metal grid; (**k**) the nanocomposite layer on a metal grid.

As methods “A” and “E” were the ones to give satisfactory results, these are illustrated in detail in Figure 1 and Figure 2, respectively.

In method “A”, the 10 *w*/*w*% PVA solution was deposited on an oxygen plasma-treated glass slide (Figure 1a) and spin-coated (Figure 1b). After the layer dried (Figure 1c), the feed mixture of the nanocomposite was added to the top (Figure 1d).

Then, the spin-coater was rotated at a slow speed (100–200 RPM, 60 s), and glycerol was added to cover the nanocomposite (Figure 1e). The speed was then increased (900–1000 RPM) (Figure 1f), and the system was illuminated by blue light for 2 min while spinning at this speed (Figure 1g).

Some samples were measured after the glycerol was dried (Figure 1h), while the others were immersed in water (room temperature, overnight) to dissolve the PVA layer as well as the remaining glycerol (Figure 1i). The floating polymerized nanocomposite layer was placed on a metal grid (Figure 1j,k).

For method “E”, the first steps (Figure 2a–d) correspond to the above-described procedure. Then, instead of adding glycerol, the speed was increased to 900–1000 RPM and the mixture was illuminated for 60 s (Figure 2e). The speed was decreased back to 100–200 RPM, glycerol was added (Figure 2f), and a second round of photopolymerization took place for 60 s as well (Figure 2g). The final steps (Figure 2h–k) are the same as the ones described in method “A”.

### 2.4. Measurements and Instruments

For optical microscopy, an Opympus BX51M microscope was used with high focal length objectives (LMPlanFl, Olympus, Japan). The magnification/numerical aperture of the used objectives were 10×/0.25, 20×/0.40, and 50×/0.50. For spectrophotometry, an Avantes Avaspec 2048-4DT spectrometer (Avantes, Apeldoorn, The Netherlands) and an Avantes Avalight DHS halogen light source (Avantes, Apeldoorn, The Netherlands) were used between 500 nm and 850 nm. The spectral slit of our spectrometer was 25 μm, resulting in a FWHM resolution of 1.4 nm. The step of the spectral data was 0.28 nm. For the evaluation of the retrieved spectra, a custom Matlab program (Matlab 2024b, MathWorks, Natick, Massachusetts, IN, USA) was written and used, and later the diagrams were plotted in OriginPro 2018 (Originlab, Northampton, MA, USA) software.

The viscosity of the UDMA-TEGDMA monomer mixture and the pure UDMA monomer was evaluated from flow curve data obtained using an MCR 102 rheometer (Anton Paar GmbH, Graz, Austria) equipped with a 25 mm parallel plate geometry. Measurements were conducted at a constant temperature of 25 °C, applying an increasing and then decreasing shear rate ramp. A Newtonian model was used for viscosity calculation, as no significant shear-thinning behavior was observed within the applied shear rate range [23].

An Alpha Step 500 surface profiler (formerly Tencor, currently KLA, Milpitas, CA, USA) was used to determine the layer thicknesses. The scan length was 5 mm with a resolution of 3.85 μm. A custom Matlab code (Matlab 2024b, MathWorks, Natick, Massachusetts, IN, USA) was written and used to evaluate the obtained data. OriginPro 2018 (Originlab, Northampton, MA, USA) was used to plot the diagrams.

## 3. Results and Discussion

Out of the five methods investigated, two (“A” and “E”) provided satisfactory results. The problem with methods “B” and “C” was that during the first spin-off, the sample was not illuminated and thus photopolymerization was not performed. As the monomer mixture has low viscosity (555.02 mPas), most of it spun off, and only a few stains remained instead of forming a continuous layer. Polymerization, even partially in the presence of oxygen, would increase the viscosity, enabling the formation of a layer.

This issue was overcome in method “D”; however, the absence of glycerol during the first polymerization led to a thin, unpolymerized resin-rich layer due to the oxygen inhibition on top of the polymerized nanocomposite layer. Glycerol was added without rotating the sample; thus, it did not spread out and contracted the monomer film. When polymerized, this caused an uneven surface.

All results described in the following sections were obtained from samples made by either method “A” or “E”.

### 3.1. Optical Microscopy

**Figure 3:**
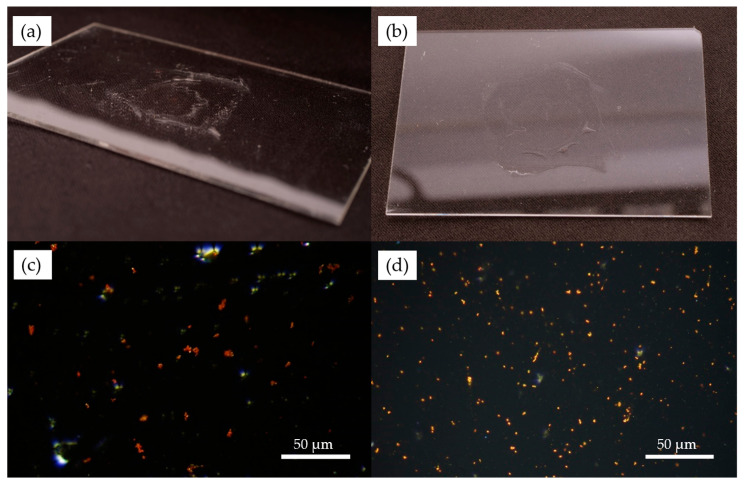
Photographs and dark-field optical microscope images of the layers after spin coating: (**a**) photograph of the UDMA-based nanocomposite layer containing 25 nm × 85 nm nanorods; (**b**) photograph of the UDMA-based nanocomposite layer containing 25 nm × 35 nm nanorods; (**c**) microscope image of the UDMA-based nanocomposite layer containing 25 nm × 85 nm nanorods; (**d**) microscope image of the UDMA-based nanocomposite layer containing 25 nm × 35 nm nanorods.

After the spin coating was finished and the glycerol dried, the samples were examined. Photographs and optical microscope images were taken, as shown in Figure 3. The layers of PVA and dimethacrylate nanocomposite spin-coated on glass slides can be observed in Figure 3a,b.

Figure 3c,d show the microscope images of the samples seen in (a) and (b), respectively. The images were taken in dark-field mode; thus, the directly transmitted light is excluded, and diffracted light is used to create the image. This method enables the observation of the characteristic light scattering caused by the nanoparticles. The color difference between Figure 3c,d is due to the size difference of the gold nanorods in the two samples.

**Figure 4:**
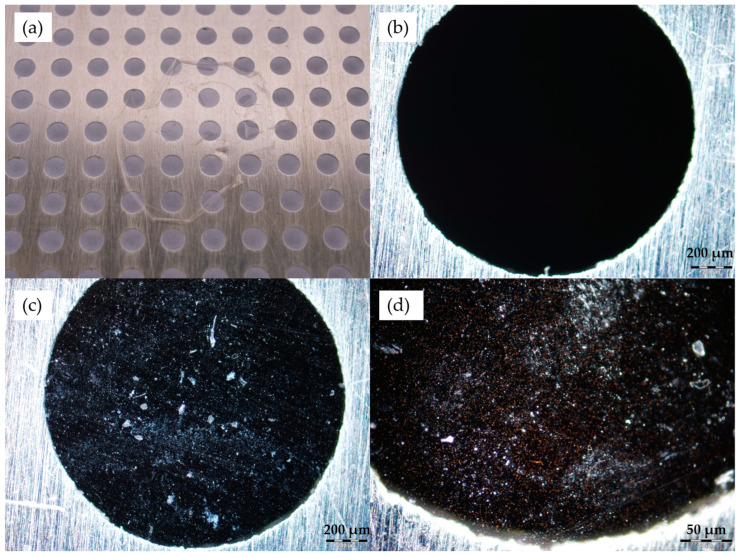
UDMA-based nanocomposite layer on the metal grid: (**a**) the nanocomposite on the grid following the dissolution of PVA; (**b**) dark-field microscopy image of the metal grid; (**c**) dark-field microscopy image of the nanocomposite layer on the grid; (**d**) dark-field microscopy image of the nanocomposite layer on the grid, showing the nanoparticles.

The nanocomposite layers were investigated after the dissolution of PVA as well. Figure 4 shows a photograph of the layer on the metal grid later used for the laser irradiation experiments (a), a microscope image of the blank grid as a reference (b), and two microscope images of the grid with the nanocomposite layer on it (c and d). Similarly to the previous images, dark-field mode was used.

In Figure 3 and Figure 4, we can observe the nanorods in the form of small clusters, which are otherwise evenly distributed in the sample. It has to be emphasized here that the distinct color of the particles confirms that they are not aggregated (i.e., they are not touching each other). The color is the result of localized surface plasmon resonance (LSPR), which defines distinct scattering spectra for the nanorods, depending on their geometry (size, shape) and the refractive index of the surrounding medium. If the particles were closer to each other than their characteristic size, their plasmon resonances would couple, causing a significant redshift of the scattering spectrum, known as the plasmon ruler effect [24]. Random particle aggregation would cause the distinct color to disappear as the size of the agglomerates surpasses the size range of LSPR conditions [25]. The fact that the absorbance/scattering peaks of the particles match the intended target application, as demonstrated with optical spectroscopy in the next section, also confirms that we see individual but clustered nanorods in the thin nanocomposite layers. In our previous work, scanning transmission electron microscopy (STEM) images were also made on similar nanocomposites (containing 25 nm × 75 nm nanorods), which confirmed the aggregation-free dispersion of the nanorods in the matrix. For more information, see [17].

### 3.2. Optical Spectroscopy

**Figure 5:**
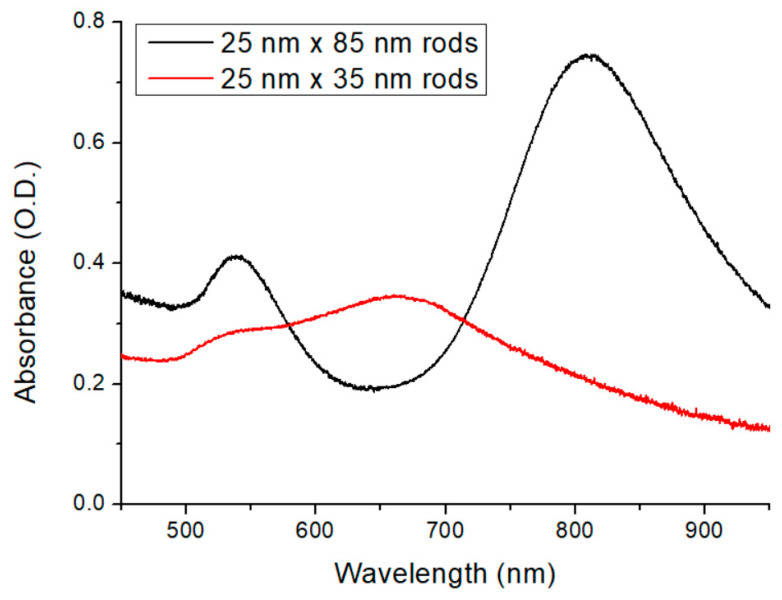
Optical spectra of the nanocomposites (black: 25 nm × 85 nm rods, red: 25 nm × 35 nm rods, respectively).

The optical spectroscopy measurements were performed on thicker nanocomposite samples (with the exact same composition as the few-micron-thick layers), as the absorbance of the thin layers was too low to be appropriately evaluated. The results are shown in Figure 5. The spectrum of the sample containing the bigger nanorods (25 nm × 85 nm) is plotted in black, while the red one represents the smaller nanorods (25 nm × 35 nm). As can be seen in Figure 5, the two nanorods give sharp LSPR peaks at different wavelengths. The longer rods have an intense longitudinal resonance mode at 800 nm and a transversal resonance mode at 540 nm, while the shorter rods have longitudinal resonance at a shorter wavelength, namely around 650 nm. (The longer rods will serve as resonant nanoantennas in the fusion targets in the NAPLIFE experiments, where the primary resonance mode is tuned to the laser emission wavelength at 795 nm, while the shorter rods will serve as non-resonant control targets [17]).

The obtained spectra correspond well with the optical microscopy investigations presented in Figure 3 and Figure 4. The sharp resonance peak corresponds well with a uniform distribution of individual nanoparticles (the full width at half-maximum is realistic considering a ~10% standard deviation in the particles’ axis lengths and the higher refractive index of the medium, which is around 1.535 [17]), with perhaps a slight amount of aggregation apparent in the slightly increased right slope of the longitudinal peak.

### 3.3. Profilometer

**Figure 6:**
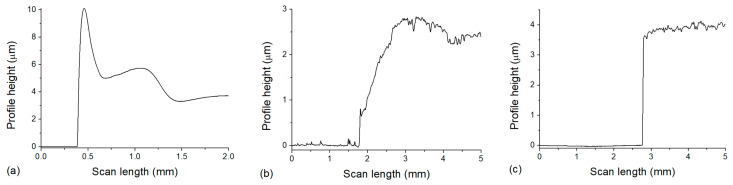
Height (thickness) of the spin-coated layers: (**a**) PVA on a glass slide; (**b**) UDMA-based nanocomposite on top of PVA (where the 0 level corresponds to the surface of the PVA layer); (**c**) UDMA-based nanocomposite on a glass slide after the dissolution of PVA.

In order to determine the thickness of the layers, measurements were performed with a profilometer. The results are shown in Figure 6. In some of the obtained profiles, a peak can be observed at the edge of the PVA layer, such as the one seen in Figure 6a, which indicates a higher ring on the edge of the PVA layer. The height of the PVA was approximately 4 µm (Figure 6a), enough to separate the nanocomposite from the glass, but not too thick, thus minimizing the dissolution time, and this thickness was uniform inside the ‘ring’ on a large (several cm^2^) area.

The results show that the nanocomposite layer’s thickness was 2.5–3 µm after photopolymerization during spin coating (Figure 6b) and approximately 3.5–4 µm after the dissolution of PVA (Figure 6c). Considering the difference in the measured values, besides experimental uncertainty that can arise from measurements on different areas, we also have to consider the water uptake of the UDMA-TEGDMA copolymer, which can possibly cause a small extent of swelling. The ester groups of TEGDMA can easily form hydrogen bonds with water molecules (TEGDMA is known to partially mix with water [26]), which is a property we utilized in order to properly wet the PVA surface during spin coating to form a uniform layer. In our previous work, differential scanning calorimetry (DSC) and thermogravimetric analysis (TGA) were performed in the same 3:1 UDMA-TEGDMA matrix [17]. An endotherm peak in the DSC at 47.7 °C confirmed the evaporation of the absorbed water from the polymer matrix in the first cycle. More interestingly, TGA indicated that the matrix can absorb water from the ambient atmosphere (e.g., even during storage). Mass losses associated with water evaporation constituted around 5% of the total mass, at temperatures well below the degradation of the polymer (which starts at 230 °C). Furthermore, Raman spectroscopy measurements also indicated a peak at 3390 cm^−1^, which can be associated with absorbed water. For more information regarding the DSC and TGA studies, see [17].

## 4. Conclusions

The present study demonstrates the possibility of creating a few-micron free-standing polymer nanocomposite film with a PVA sacrificial layer by applying a spin-coating process. In particular, the results showed that the spin-coating technique, the photopolymerization conditions, and the sequence of the individual steps strongly influence the composite layer’s thickness and integrity. In addition, the optimal selection of the sacrificial layer material is also essential for film formation. PVA was found to be suitable as the sacrificial layer due to its good water solubility, good film-forming properties, and interaction with dimethacrylate monomers. The interaction between the OH groups of PVA and the ester bonds of dimethacrylates contributes to the continuous film formation of the dimethacrylate resin mixture. Dark-field optical microscopy images demonstrated the presence of the nanoparticles in the UDMA nanocomposite layer, suggesting that the technology employed is suitable for these purposes. In accordance with the spectra, nanorods of different sizes scatter light differently, resulting in two distinct colors in the microscope images. This also confirms the uniform, aggregation-free distribution of nanorods in the matrix. The 2–3 µm UDMA-TEGDMA film thickness was the lowest that could be attained, as increasing the rotation speed resulted in the loss of layer integrity or even the disappearance of the layer. This few-micron thickness is also significantly lower than the usual oxygen inhibition layer thickness, which is around 4–40 µm for such resins. By fine-tuning the technology, further improvements could be made for more demanding applications.

## Figures and Tables

**Table 1:** Parameters of the tested spin-coating methods with five different approaches.

Method “A”	Method “B”	Method “C”	Method “D”	Method “E”
Nanocomposite spin-up (100–200 RPM, 30 s)	Nanocomposite spin-up (100–200 RPM, 30 s)	Nanocomposite spin-up (100–200 RPM, 30 s)	Nanocomposite spin-up (100–200 RPM, 30 s)	Nanocomposite spin-up (100–200 RPM, 30 s)
Deposition and spin-up of glycerol (100–200 RPM, 30 s)	Spin-off (900–1000 RPM, 60 s)	Spin-off (900–1000 RPM, 60 s)	Photopolymerization during spin-off (900–1000 RPM, 60 s)	Photopolymerization during spin-off (900–1000 RPM, 60 s)
Photopolymerization during spin-off (900–1000 RPM, 120 s)	Deposition of glycerol, photopolymerization (0 RPM, 120 s)	Deposition and spin-up of glycerol, photopolymerization (100–200 RPM, 120 sec)	Deposition of glycerol, photopolymerization (0 RPM, 60 s)	Deposition and spin-up of glycerol, photopolymerization (100–200 RPM, 60 s)

## Data Availability

The original contributions presented in this study are included in the article. Further inquiries can be directed to the corresponding author.

## Abbreviations

The following abbreviations are used in this manuscript:

Au-DDT	dodecanethiol-capped gold nanorods
CQ	camphorquinone
DC	degree of conversion
DSC	differential scanning calorimetry
EDAB	ethyl-4-dimethylaminobenzoate
LSPR	localized surface plasmon resonance
NAPLIFE	NanoPlasmonic Laser Induced Fusion Experiment
OIL	oxygen inhibition layer
PVA	polyvinyl alcohol
RPM	rotation per min
STEM	scanning transmission electron microscopy
TEGDMA	triethylene glycol dimethacrylate
TGA	thermogravimetric analysis
UDMA	urethane dimethacrylate
